# Crystal structure of iron(III) perchlorate nona­hydrate

**DOI:** 10.1107/S1600536814024295

**Published:** 2014-11-12

**Authors:** Erik Hennings, Horst Schmidt, Wolfgang Voigt

**Affiliations:** a TU Bergakademie Freiberg, Institute of Inorganic Chemistry, Leipziger Strasse 29, D-09596 Freiberg, Germany

**Keywords:** crystal structure, low-temperature salt hydrates, perchlorate hydrates, iron perchlorate, Mars

## Abstract

Since the discovery of perchlorate salts on Mars and the known occurrence of ferric salts in the regolith, there is a distinct possibility that the title compound could form on the surface of Mars. [Fe(H_2_O)_6_](ClO_4_)_3_·3H_2_O was crystallized from aqueous solutions at low temperatures according to the solid–liquid phase diagram. It consists of Fe(H_2_O)_6_ octa­hedra (point group symmetry -3.) and perchlorate anions (point group symmetry .2) as well as non-coordinating water mol­ecules, as part of a second hydrogen-bonded coordination sphere around the cation. The perchlorate appears to be slightly disordered, with major–minor component occupancies of 0.773 (9):0.227 (9).

## Abstract 2

Since the discovery of perchlorate salts on Mars and the known occurrence of ferric salts in the regolith, there is a distinct possibility that the title compound could form on the surface of Mars. [Fe(H_2_O)_6_](ClO_4_)_3_·3H_2_O was crystallized from aqueous solutions at low temperatures according to the solid–liquid phase diagram.

## Chemical context   

Since the discovery of perchlorate salts on the surface of Mars during the Phoenix expedition (Hecht *et al.*, 2009 ▶; Davila *et al.*, 2013 ▶; Kerr, 2013 ▶; Marion *et al.*, 2010 ▶; Navarro-González *et al.*, 2010 ▶), inter­est in the solubility and crystal structures of the perchlorate hydrate phases became more important (Chevrier, Hanley & Altheide, 2009 ▶; Catling *et al.*, 2010 ▶). Based on the red color of the planet, one can expect different iron phases, such as perchlorate and sulfate, to be important constituents of the regolith (Chevrier, Ulrich & Altheide, 2009 ▶; Chevrier & Altheide, 2008 ▶; Hennings *et al.*, 2013 ▶). While investigating the solubility of ferric perchlorate, we obtained the nona­hydrate as a stable phase in the binary salt–water system.

## Structural commentary   

**Figure 1:**
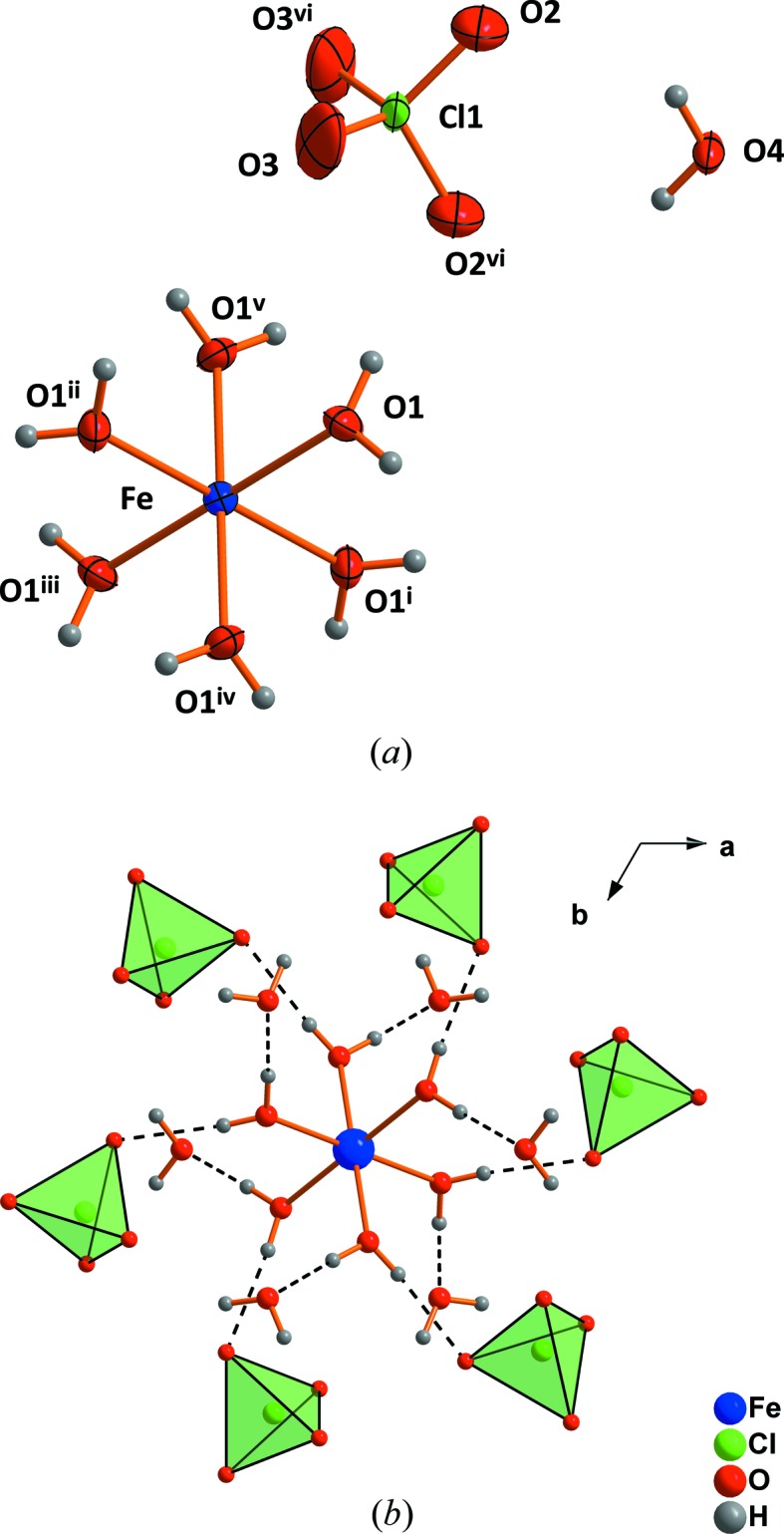
The mol­ecular units (*a*) and second coordination sphere (*b*) of ferric perchlorate nona­hydrate. Dashed lines indicate hydrogen bonds. Displacement ellipsoids are drawn at the 50% probability limit. The minor disorder component of the ClO_4_ tetrahedron has been omitted. [Symmetry codes: (i) *x* − *y*, *x*, 1 − *z*; (ii) −*x* + *y*, −*x*, *z*; (iii) −*x*, −*y*, 1 − *z*; (iv) −*y*, x − *y*, *z*; (v) *y*, −*x* + *y*, 1 − *z*; (vi) 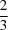 − *x*, 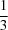 − *x* + *y*, 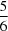 − *z*.]

**Figure 2:**
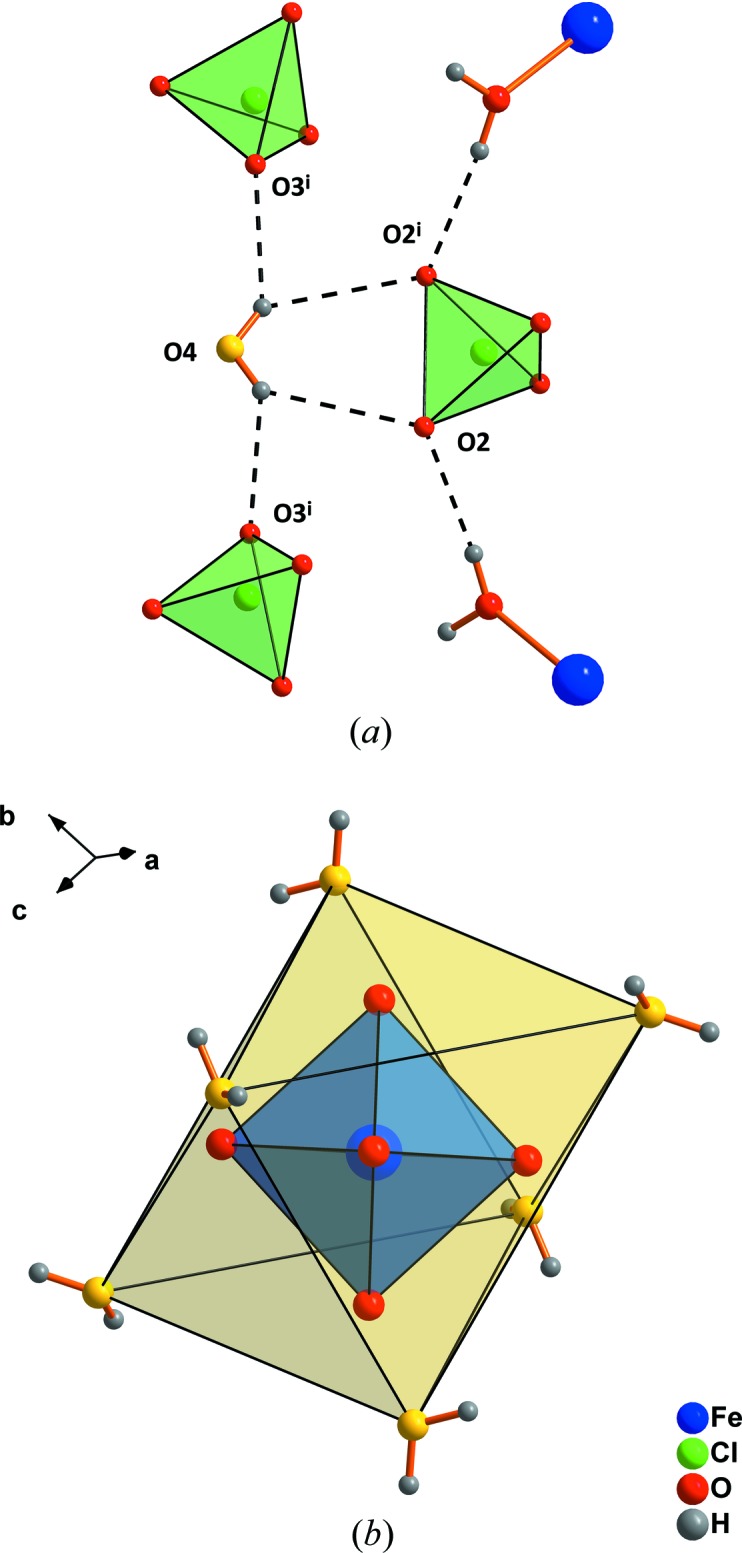
The connection scheme of water mol­ecules of the second coordination sphere by hydrogen bonds (*a*) and the formation of a secondary hydration shell (yellow) around the cations (*b*). The minor disorder component of the ClO_4_ tetrahedron has been omitted for clarity. Dashed lines indicate hydrogen bonds. [Symmetry code: (i) 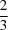 − *x*, 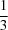 − *x* + *y*, 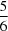 − *z*.]

The central Fe atom is situated on a threefold inversion axis and is octa­hedrally coordinated by six water mol­ecules in the first, and by six water mol­ecules as well as six perchlorate tetra­hedra in the second coordination spheres (Fig. 1 ▶). The water mol­ecules of the second coordination sphere (O4 and symmetry equivalents) are connected to perchlorate tetra­hedra (Fig. 2 ▶ *a*) *via* hydrogen bonds (Table 1 ▶). Six O4-water mol­ecules form a second, larger octa­hedron outside the octa­hedron of the first coordination shell (Fig. 2 ▶ *b*). The perchlorate anion, situated on a twofold rotation axis, appears to be slightly disordered, with major:minor component occupancies of 0.773 (9):0.227 (9).

## Supra­molecular features   

**Figure 3:**
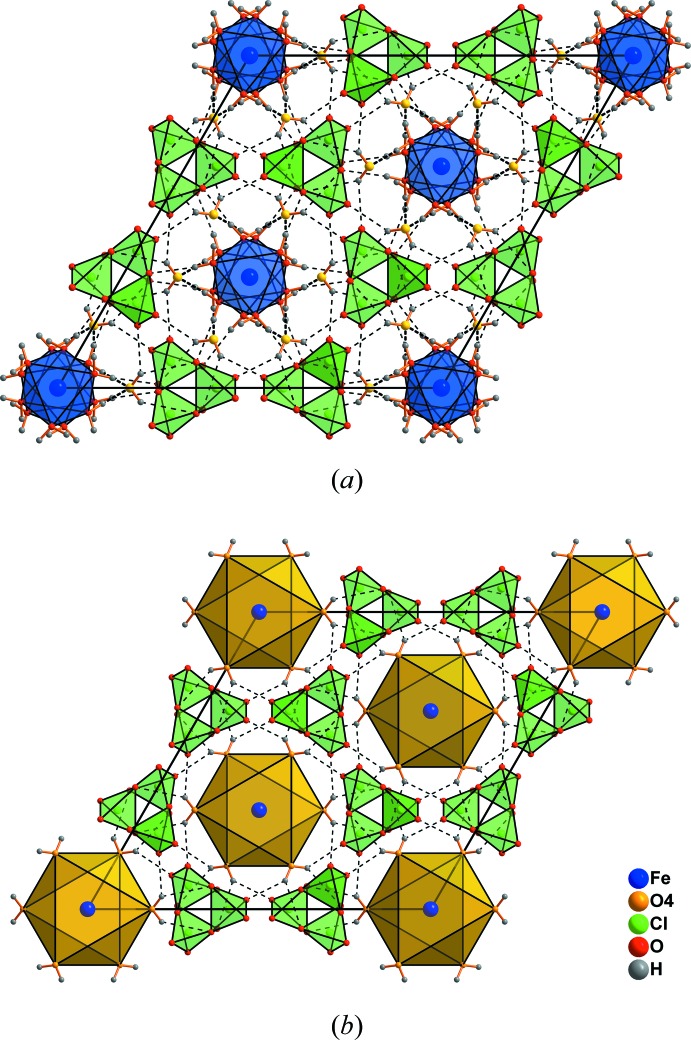
The unit cell of iron(III) perchlorate nona­hydrate with coordination polyhedra of the first (*a*) and second (*b*) coordination sphere. The minor disorder component of the ClO_4_ tetrahedron has been omitted for clarity. Dashed lines indicate hydrogen bonds.

From the unit cell of ferric perchlorate nona­hydrate (Fig. 3 ▶ *a*), it is obvious that the O4 atoms form a secondary hydration shell around the Fe(H_2_O)_6_ units. This becomes clearer when drawing the second octa­hedra as water coordination polyhedra (yellow, Fig. 3 ▶ *b*). The water mol­ecules of the second coordination sphere are closer [4.143 (4) Å] to the Fe atom than the perchlorate tetra­hedra [4.271 (4) Å].

## Database survey   

For crystal structure determination of other perchlorate nona­hydrates, see: Davidian *et al.* (2012 ▶) for the Al, Ga and Sc salts and Hennings *et al.* (2014 ▶) for the strontium salt. For crystal structure determinations of other Fe^III^ salts with a high water content, see: Schmidt *et al.* (2013 ▶); Lindstrand (1936 ▶).

## Synthesis and crystallization   

Iron(III) perchlorate nona­hydrate crystallized from an aqueous solution of 54.41 wt% Fe(ClO_4_)_3_ thermostated at 263 K after 2 d. To prepare this solution, ferric perchlorate nona­hydrate (Fluka, pure) was used. The content of Fe^III^ ions was analysed using gravimetric analysis by precipitation with ammonia. All crystals are stable in their saturated solution over a period of at least four weeks.

The samples were stored in a freezer or a cryostat at low temperatures. The crystals were separated and embedded in perfluorinated ether for X-ray diffraction analysis

## Refinement   

Crystal data, data collection and structure refinement details are summarized in Table 2 ▶. The H atoms were placed in the positions indicated by difference Fourier maps. No further constraints were applied.

## Supplementary Material

Crystal structure: contains datablock(s) I. DOI: 10.1107/S1600536814024295/pk2533sup1.cif

Structure factors: contains datablock(s) I. DOI: 10.1107/S1600536814024295/pk2533Isup2.hkl

CCDC reference: 1032663

Additional supporting information:  crystallographic information; 3D view; checkCIF report

## Figures and Tables

**Table 1:** Hydrogen-bond geometry (, )

*D*H*A*	*D*H	H*A*	*D* *A*	*D*H*A*
O1H1*A*O2^i^	0.83(5)	1.92(5)	2.745(5)	174(4)
O1H1*A*O2^i^	0.83(5)	2.33(5)	3.153(14)	170(4)
O1H1*A*O3	0.83(5)	2.27(5)	2.864(17)	129(4)
O1H1*B*O4^ii^	0.82(5)	1.83(5)	2.642(3)	173(4)
O4H4O2	0.84(4)	2.39(4)	3.073(5)	139(4)
O4H4O3^iii^	0.84(4)	2.13(4)	2.796(4)	136(4)
O4H4O2	0.84(4)	2.13(5)	2.812(17)	138(4)
O4H4O3^iii^	0.84(4)	2.12(4)	2.708(12)	127(4)

Symmetry codes: (i) 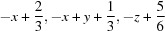; (ii) 
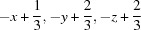
; (iii) 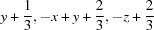.

**Table 2:** Experimental details

Crystal data	
Chemical formula	[Fe(H_2_O)_6_](ClO_4_)_3_3H_2_O
*M*_r_	516.34
Crystal system, space group	Trigonal, *R* 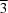 *c*:*H*
Temperature (K)	100
*a*, *c* ()	16.1930(15), 11.2421(11)
*V* (^3^)	2552.9(5)
*Z*	6
Radiation type	Mo *K*
(mm^1^)	1.46
Crystal size (mm)	0.54 0.37 0.19

Data collection	
Diffractometer	STOE IPDS 2T
Absorption correction	Integration (Coppens, 1970 ▶)
*T*_min_, *T*_max_	0.531, 0.755
No. of measured, independent and observed [*I* > 2(*I*)] reflections	8865, 659, 641
*R*_int_	0.075
(sin /)_max_ (^1^)	0.650

Refinement	
*R*[*F* ^2^ > 2(*F* ^2^)], *wR*(*F* ^2^), *S*	0.041, 0.092, 1.11
No. of reflections	658
No. of parameters	60
H-atom treatment	All H-atom parameters refined
_max_, _min_ (e ^3^)	0.64, 0.80

Computer programs: *X-AREA* and *X-RED* (Stoe Cie, 2009 ▶), *SHELXS97* and *SHELXL2012* (Sheldrick, 2008 ▶), *DIAMOND* (Brandenburg, 2006 ▶) and *publCIF* (Westrip, 2010 ▶).

## supplementary crystallographic information

### Crystal data

**Table:**

[Fe(H_2_O)_6_](ClO_4_)_3_·3H_2_O	*D*_x_ = 2.015 Mg m^−^^3^
*M**_r_* = 516.34	Mo *K*α radiation, λ = 0.71073 Å
Trigonal, *R*3*c*:*H*	Cell parameters from 47287 reflections
*a* = 16.1930 (15) Å	θ = 7.0–29.7°
*c* = 11.2421 (11) Å	µ = 1.46 mm^−^^1^
*V* = 2552.9 (5) Å^3^	*T* = 100 K
*Z* = 6	Needle, colorless
*F*(000) = 1578	0.54 × 0.37 × 0.19 mm

### Data collection

**Table:**

STOE IPDS 2T diffractometer	659 independent reflections
Radiation source: fine-focus sealed tube	641 reflections with *I* > 2σ(*I*)
Detector resolution: 6.67 pixels mm^-1^	*R*_int_ = 0.075
rotation method scans	θ_max_ = 27.5°, θ_min_ = 2.5°
Absorption correction: integration (Coppens, 1970)	*h* = −20→20
*T*_min_ = 0.531, *T*_max_ = 0.755	*k* = −20→20
8865 measured reflections	*l* = −14→14

### Refinement

**Table:**

Refinement on *F*^2^	0 restraints
Least-squares matrix: full	Hydrogen site location: difference Fourier map
*R*[*F*^2^ > 2σ(*F*^2^)] = 0.041	All H-atom parameters refined
*wR*(*F*^2^) = 0.092	*w* = 1/[σ^2^(*F*_o_^2^) + (0.0269*P*)^2^ + 23.9134*P*] where *P* = (*F*_o_^2^ + 2*F*_c_^2^)/3
*S* = 1.11	(Δ/σ)_max_ < 0.001
658 reflections	Δρ_max_ = 0.64 e Å^−^^3^
60 parameters	Δρ_min_ = −0.80 e Å^−^^3^

### Special details

**Table:**

Geometry. All esds (except the esd in the dihedral angle between two l.s. planes) are estimated using the full covariance matrix. The cell esds are taken into account individually in the estimation of esds in distances, angles and torsion angles; correlations between esds in cell parameters are only used when they are defined by crystal symmetry. An approximate (isotropic) treatment of cell esds is used for estimating esds involving l.s. planes.

### Fractional atomic coordinates and isotropic or equivalent isotropic displacement parameters (Å^2^)

**Table:**

	*x*	*y*	*z*	*U*_iso_*/*U*_eq_	Occ. (<1)
Fe1	0.0000	0.0000	0.5000	0.0188 (3)	
O1	0.07420 (15)	0.11666 (15)	0.3991 (2)	0.0252 (5)	
O4	0.3333	0.47858 (17)	0.4167	0.0247 (6)	
Cl1	0.3333	0.2540 (19)	0.4167	0.0329 (3)	0.773 (9)
O2	0.4134 (3)	0.3438 (3)	0.3808 (4)	0.0342 (8)	0.773 (9)
O3	0.3070 (3)	0.1914 (3)	0.3110 (4)	0.0481 (12)	0.773 (9)
Cl1'	0.3333	0.254 (7)	0.4167	0.0329 (3)	0.227 (9)
O2'	0.3946 (11)	0.3499 (13)	0.3527 (16)	0.0342 (8)	0.227 (9)
O3'	0.2699 (12)	0.1716 (10)	0.3602 (15)	0.0481 (12)	0.227 (9)
H1A	0.129 (3)	0.158 (3)	0.417 (4)	0.047 (12)*	
H1B	0.048 (3)	0.138 (3)	0.357 (4)	0.051 (13)*	
H4	0.375 (3)	0.468 (3)	0.388 (4)	0.054 (13)*	

### Atomic displacement parameters (Å^2^)

**Table:**

	*U*^11^	*U*^22^	*U*^33^	*U*^12^	*U*^13^	*U*^23^
Fe1	0.0157 (3)	0.0157 (3)	0.0251 (5)	0.00784 (15)	0.000	0.000
O1	0.0165 (10)	0.0195 (10)	0.0341 (11)	0.0050 (8)	−0.0004 (8)	0.0045 (8)
O4	0.0269 (15)	0.0164 (9)	0.0344 (16)	0.0135 (8)	0.0102 (12)	0.0051 (6)
Cl1	0.0221 (5)	0.0137 (4)	0.0659 (8)	0.0110 (2)	−0.0180 (5)	−0.0090 (2)
O2	0.0180 (19)	0.0381 (15)	0.051 (2)	0.0174 (13)	−0.0027 (15)	0.0098 (16)
O3	0.045 (3)	0.0345 (19)	0.067 (3)	0.0210 (19)	−0.0055 (19)	−0.0276 (19)
Cl1'	0.0221 (5)	0.0137 (4)	0.0659 (8)	0.0110 (2)	−0.0180 (5)	−0.0090 (2)
O2'	0.0180 (19)	0.0381 (15)	0.051 (2)	0.0174 (13)	−0.0027 (15)	0.0098 (16)
O3'	0.045 (3)	0.0345 (19)	0.067 (3)	0.0210 (19)	−0.0055 (19)	−0.0276 (19)

### Geometric parameters (Å, º)

**Table:**

Fe1—O1^i^	2.007 (2)	Cl1—O2	1.439 (18)
Fe1—O1^ii^	2.007 (2)	Cl1—O3^vi^	1.479 (17)
Fe1—O1^iii^	2.007 (2)	Cl1—O3	1.479 (17)
Fe1—O1^iv^	2.007 (2)	Cl1'—O3'^vi^	1.37 (6)
Fe1—O1^v^	2.007 (2)	Cl1'—O3'	1.37 (6)
Fe1—O1	2.007 (2)	Cl1'—O2'^vi^	1.54 (7)
Cl1—O2^vi^	1.439 (18)	Cl1'—O2'	1.54 (7)
			
O1^i^—Fe1—O1^ii^	180.00 (9)	O1^v^—Fe1—O1	180.00 (10)
O1^i^—Fe1—O1^iii^	91.19 (9)	O2^vi^—Cl1—O2	112 (2)
O1^ii^—Fe1—O1^iii^	88.81 (9)	O2^vi^—Cl1—O3^vi^	105.8 (3)
O1^i^—Fe1—O1^iv^	88.81 (9)	O2—Cl1—O3^vi^	109.42 (19)
O1^ii^—Fe1—O1^iv^	91.19 (9)	O2^vi^—Cl1—O3	109.42 (19)
O1^iii^—Fe1—O1^iv^	180.00 (10)	O2—Cl1—O3	105.8 (3)
O1^i^—Fe1—O1^v^	91.19 (9)	O3^vi^—Cl1—O3	114 (2)
O1^ii^—Fe1—O1^v^	88.81 (9)	O3'^vi^—Cl1'—O3'	106 (7)
O1^iii^—Fe1—O1^v^	91.19 (9)	O3'^vi^—Cl1'—O2'^vi^	124.0 (13)
O1^iv^—Fe1—O1^v^	88.81 (9)	O3'—Cl1'—O2'^vi^	105.4 (10)
O1^i^—Fe1—O1	88.81 (9)	O3'^vi^—Cl1'—O2'	105.4 (10)
O1^ii^—Fe1—O1	91.19 (9)	O3'—Cl1'—O2'	124.0 (13)
O1^iii^—Fe1—O1	88.81 (9)	O2'^vi^—Cl1'—O2'	94 (6)
O1^iv^—Fe1—O1	91.19 (9)		

Symmetry codes: (i) *y*, −*x*+*y*, −*z*+1; (ii) −*y*, *x*−*y*, *z*; (iii) *x*−*y*, *x*, −*z*+1; (iv) −*x*+*y*, −*x*, *z*; (v) −*x*, −*y*, −*z*+1; (vi) −*x*+2/3, −*x*+*y*+1/3, −*z*+5/6.

### Hydrogen-bond geometry (Å, º)

**Table:**

*D*—H···*A*	*D*—H	H···*A*	*D*···*A*	*D*—H···*A*
O1—H1*A*···Cl1	0.83 (5)	2.86 (5)	3.642 (2)	157 (4)
O1—H1*A*···O2^vi^	0.83 (5)	1.92 (5)	2.745 (5)	174 (4)
O1—H1*A*···O2′^vi^	0.83 (5)	2.33 (5)	3.153 (14)	170 (4)
O1—H1*A*···O3′	0.83 (5)	2.27 (5)	2.864 (17)	129 (4)
O1—H1*B*···O4^vii^	0.82 (5)	1.83 (5)	2.642 (3)	173 (4)
O4—H4···O2	0.84 (4)	2.39 (4)	3.073 (5)	139 (4)
O4—H4···O3^viii^	0.84 (4)	2.13 (4)	2.796 (4)	136 (4)
O4—H4···O2′	0.84 (4)	2.13 (5)	2.812 (17)	138 (4)
O4—H4···O3′^viii^	0.84 (4)	2.12 (4)	2.708 (12)	127 (4)

Symmetry codes: (vi) −*x*+2/3, −*x*+*y*+1/3, −*z*+5/6; (vii) −*x*+1/3, −*y*+2/3, −*z*+2/3; (viii) *y*+1/3, −*x*+*y*+2/3, −*z*+2/3.

## References

[bb1] Brandenburg, K. (2006). *DIAMOND*. Crystal Impact GbR, Bonn, Germany.

[bb2] Catling, D. C., Claire, M. W., Zahnle, K. J., Quinn, R. C., Clark, B. C., Hecht, M. H. & Kounaves, S. (2010). *J. Geophys. Res.* **115**, 1–15.

[bb3] Chevrier, V. F. & Altheide, T. S. (2008). *Geophys. Res. Lett.* **35**, 1–5

[bb4] Chevrier, V. F., Hanley, J. & Altheide, T. S. (2009). *Geophys. Res. Lett.* **36**, 1–6.

[bb5] Chevrier, V. F., Ulrich, R. & Altheide, T. S. (2009). *J. Geophys. Res.* **114**, 1–11.

[bb6] Coppens, P. (1970). *Crystallographic Computing*, edited by F. R. Ahmed, S. R. Hall & C. P. Huber, pp. 255–270. Copenhagen: Munksgaard.

[bb7] Davidian, A. G., Pestova, O. N., Starova, G. L., Gurzhii, V. V., Myund, L. A. & Khripun, M. K. (2012). *Russ. J. Gen. Chem.* **82**, 612–625.

[bb8] Davila, A. F., Willson, D., Coates, J. D. & McKay, C. P. (2013). *Int. J. Astrobiology*, **12**, 321–325.

[bb9] Hecht, M. H., Kounaves, S. P., Quinn, R. C., West, S. J., Young, S. M. M., Ming, D. W., Catling, D. C., Clark, B. C., Boynton, W. V., Hoffman, J., Deflores, L. P., Gospodinova, K., Kapit, J. & Smith, P. H. (2009). *Science*, **325**, 64–67. 10.1126/science.1172466 19574385

[bb10] Hennings, E., Schmidt, H. & Voigt, W. (2014). *Acta Cryst* E**70**, 510–514. 10.1107/S1600536814024726 PMC4257379 25552979

[bb11] Hennings, E., Zürner, P., Schmidt, H. & Voigt, W. (2013). *Icarus*, **226**, 268–271.

[bb12] Kerr, R. A. (2013). *Science*, **340**, p. 138. 10.1126/science.340.6129.138-b 23580505

[bb13] Lindstrand, F. (1936). *Z. Anorg. Allg. Chem.* **230**, 187–208.

[bb14] Marion, G. M., Catling, D. C., Zahnle, K. J. & Claire, M. W. (2010). *Icarus*, **207**, 678–685.

[bb15] Navarro-González, R., Vargas, E., de la Rosa, J., Raga, A. C. & McKay, C. P. (2010). *J. Geophys. Res.* **115**, 1–11.

[bb16] Schmidt, H., Hennings, E., Zürner, P. & Voigt, W. (2013). *Acta Cryst.* C**69**, 330–333. 10.1107/S0108270113005969 23579698

[bb17] Sheldrick, G. M. (2008). *Acta Cryst.* A**64**, 112–122. 10.1107/S0108767307043930 18156677

[bb18] Stoe & Cie (2009). *X-AREA* and *X-RED*. Stoe & Cie, Darmstadt, Germany.

[bb19] Westrip, S. P. (2010). *J. Appl. Cryst.* **43**, 920–925.

